# The global burden of lower urinary tract symptoms suggestive of benign prostatic hyperplasia: A systematic review and meta-analysis

**DOI:** 10.1038/s41598-017-06628-8

**Published:** 2017-08-11

**Authors:** Shaun Wen Huey Lee, Esther Mei Ching Chan, Yin Key Lai

**Affiliations:** 1 School of Pharmacy, Monash University Malaysia, Bandar Sunway, 47500 Malaysia; 2 School of Pharmacy, UCSI Universit, Taman Connaught, 56000 Malaysia

## Abstract

Benign prostatic hyperplasia is a common non-malignant condition among older men, but the epidemiology is poorly characterised. We summarised and determined the global prevalence of benign prostatic hyperplasia. A systematic search on PubMed, EMBASE and CENTRAL was performed up until 31^st^ July 2016. Studies that described the epidemiology of benign prostatic hyperplasia were included and cumulative plots of prevalence estimates were calculated. A total of 31 prevalence rate estimates from 25 countries were identified. The combined prevalence estimates showed that the lifetime prevalence of BPH was 26.2% (95% CI: 22.8–29.6%). We found that there was an increasing prevalence of BPH with age. However, we found no significant difference between (a) rural, urban or mixed sites, (b) different countries, (c) respondent representativeness. (d) sample size or (e) study quality. We also found no significant change in the prevalence over the past 20 years. While there is substantial variation between sites estimates, results suggest that nearly 1 in 4 men will suffer from BPH over their lifetime. The study revealed there are significant gaps in knowledge, which provides opportunities for future research to further enrich the epidemiological landscape with data.

## Introduction

Benign prostatic hyperplasia (BPH) is one of the most common urological diseases among men^1^. It is characterised by a benign overgrowth of prostatic tissue around the urethra which ultimately constricts the urethral opening, resulting in lower urinary tract symptoms (LUTS). Symptoms associated with LUTS include urgency, frequency, nocturia, incomplete urination, and weak urinary stream^2^. If left untreated, complications such as urinary retention, renal insufficiency and bladder stone can occur, requiring surgical intervention. BPH has also been associated with other medical morbidities, such as increased risk of falls^3^, reduced quality of life^4^ as well as increased annual healthcare cost^5^. As such, an understanding of the epidemiology of BPH is essential in health service planning as well as risk factor epidemiology.

Several longitudinal population based studies have provided some insights into the risk of BPH symptoms and progression. For example, the Olhmsted County study found that there was an increasing prevalence of moderate to severe symptoms of LUTS in men, increasing from 13% in men aged 40 to 49 years and 28% in men older than 70 years^6^. This number is expected to increase over the next few decades, mainly due to the increase in number of geriatrics as well as life expectancy. Several scholarly narrative reviews have been recently published in the past decade on the prevalence of BPH, but there has been substantial variation in the reported prevalence, ranging from 14% to 30% for men aged 50 or older depending on the definition used^7–9^. Many factors are thought to influence the clinical profile of patients presenting with BPH, including the differences in treatment culture, health service utilisation, degree of urbanisation and ethnicity.

Unfortunately, much of these data is heterogeneous, with variable methodological quality. In addition, most of these studies have yet to be subjected to the rigour of a systematic review and meta-analysis. This lack of synthesis makes it difficult for healthcare professionals and government officials to apply these any findings in their daily practice and public health planning. In the present study, we conducted a systematic review and meta-analysis to provide an initial baseline estimate of the prevalence of BPH in men worldwide and determine the various factors that are thought to influence the variations in reported prevalence.

## Results

**Figure 1:**
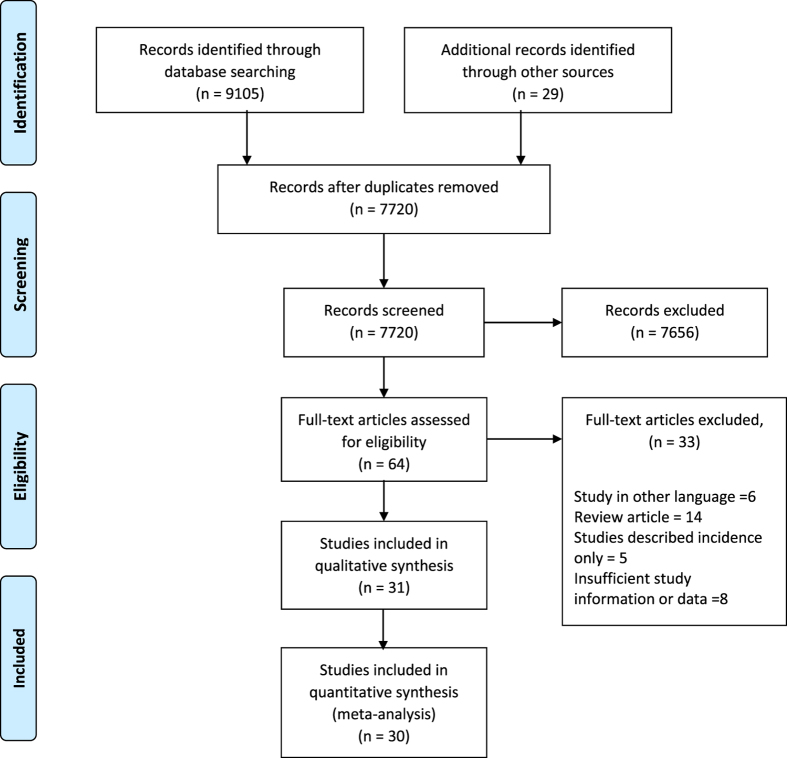
Flow of the study.

This systematic review identified a total of 31 studies^6, 10–39^ with sufficiently suitable data (Fig. 1) obtained from 25 countries. They comprised of fourteen published population based studies, thirteen community based studies, as well as four published studies estimated from clinic based cohorts. Twelve took place in Asia, 11 in Western Europe, 6 in North America and 2 in Australia and New Zealand and 1 in Africa. The number of participants per study varied considerably, ranging from 288 to 26,446 participants and all participants included were aged 30 years and above (Table 1).

**Table 1:** Summary of studies which examined the prevalence of benign prostatic hyperplasia.

Study	Country	Study year	Population source	Survey (response rate)	Diagnosis/definition of BPH	BPH prevalence
Sommer *et al*.	Denmark	1990	572 men aged 20–79 yrs from National Register (67%)	Population based, random sample, cross-sectional, postal questionnaire	Patients with obstructive and irritative symptoms based upon the modified Madsen questionnaire with total score >9	By age: 2% for 30–39; 7% for 40–49; 18% for 50–59; 23% for 60–69
Garraway *et al*.	Scotland	1991	705 men aged 40–79 yr (77%) from Bridge of Allan town	Population based, cross sectional, self-administered survey with uroflowmetry	Enlargement of prostate gland >20 g, with the presence of symptoms of urinary dysfunction (score >11) and/or Q_max_ < 15 ml/s, with no known radiological or histological evidence of prostatic malignancy	Overall: 25.3% By age: 13.8% for 40–49; 23.7% for 50–59; 43.0% for 60–69; 40.0% for 70–79
McKelvie *et al*.	Scotland	1993	2,497 men aged 40–79 yr (65.1%) from Forth Valley, Stirling	Population based, cross sectional, survey with uroflowmetry	Transrectal ultrasound measured prostate >20 g	Overall: 1 in every 4 men By age: 12.9% for 40–49; 40.4% for 70–79
Chute *et al*.	USA	1993	3854 men aged 40–79 yrs from Olmsted County (71%)	Population based, stratified sample, interview	Obstructive symptom scores mapped to AUA symptom index with a score >7	Overall: By age: 26% for 40–49; 33% for 50–59; 41% for 60–69; 46% for 70–79
Sagnier *et al*.	France	1994	2011 men aged 50–80 yrs stratified by regions and sample size	Population based, cross sectional, survey	IPSS total score >7	Overall: 14.2% By age: 8% for 50–59; 14% for 60–69; 27% for 70–79
Norman *et al*.	Canada	1994	508 men aged 50 yr and above stratified by province	Population based, cross sectional, telephone survey	Moderate or severe symptom on modified AUA symptom index (total score >7)	Overall: 23% By age: 15% for 50–59; 27% for 60–69; 31% for >70
Hunter *et al*.	Britain	1994	1480 men aged >55 yr (78%) from North West Thames region	Population based, cross sectional, postal questionnaire	Moderate or severe symptom (total score >9) on modified AUA symptom index	Overall: 20.4% By age: 16.2% for 55–59; 19.4% for 60–64; 20.9% for 65–69; 25.9% for 70–74; 20.3% for 75–79; 25.5% for 80–84; 11.9% for >85
Bosch *et al*.	Netherlands	1995	502 men aged 55–74 yrs from Rotterdam	Community based, cross sectional, self-administered questionnaire	Moderate or severe symptoms (total score >7) on the IPSS, urinary flow and prostate size	Overall: 30% By age: 26% for 55–59; 30% for 60–64; 30% for 65–69; 36% for 70–74
Tsukamoto *et al*.	Japan	1995	682 men aged 40–79 yrs from southwest Hokkaido (42.4%)	Community based, cross sectional, self-administered questionnaire with physical examination	Moderate or severe symptoms (total score >7) on the IPSS	Overall: 36.6% By age: 47% for 40–49; 44% for 50–59; 52% for 60–69; 63% for 70–79
Nacey *et al*.	New Zealand	1995	515 men aged from >40 yrs from Wellington (64%)	Community based, cross sectional, questionnaire with uroflowmetry	IPSS total score >7	Overall: 23.0% By age: 12.9% for 40–49; 22.3% for 50–59; 33.7% for 60–69; 33.3% for >70
Ukimura *et al*.	Japan	1996	961 men aged 55–87 yr from 9 rural towns in Kyoto, Shiga and Hokkaido Prefectures	Community based, cross sectional, self-administered questionnaire with physical examination	A more round appearance with greater antero-posterior diameter evaluated with transrectal ultrasonographic	Overall: 27.6% By age: 9.4% for 55–59; 15.2% for 60–64; 21.4% for 65–69; 26.9% for 70–74; 31.6% 75–79; 30.4% for >80
Hunter *et al*.	Spain	1996	2939 men aged >50 yr (68.1%) from Madrid	Population based, cross sectional, interview	Moderate or severe symptoms (total score >7) on the IPSS	Overall: 30.4% By age: 18.5% for 50–54; 19.2% for 55–59; 27.8% for 60–64; 33.6% for 65–69; 36.2% for 70–74; 45.5% 75–79; 40.0% for 80–84; 46.3% for >85
Simpson *et al*.	Scotland	1996	597 men aged 40–79 yr (55%)	Community based, cross sectional, self-administered survey with uroflowmetry	Enlargement of prostate gland >20 g, with the presence of symptoms of urinary dysfunction (score >11) and/or Q_max_ < 15 ml/s, with no known radiological or histological evidence of prostatic malignancy	Overall: 20.1% By age: 8.3% for 40–49; 17.6% for 50–59; 33.3% for 60–69; 32.1% for 70–79
Lee *et al*.	Korea	1997	519 men aged >50 yrs from Yonchon county	Community based, cross sectional, survey	IPSS total score >7	Overall: 23.2% By age: 17.7% for 50–59; 23.3% for 60–69; 35.3% for >70
Homma *et al.*	Japan,China, Korea, Taiwan, Phillipines, Thailand, Singapore, Pakistan, India, Australia	1997	7588 men aged 40-79 yr from 9 countries	Community based, cross sectional, selfadministered questionnaire	Moderate or severe symptoms (total score>7) on the IPSS	Asia By age: 18% for 40–49; 29% for 50–59; 40% for 60–69; 56% for 70–79
						Australia By age: 36% for 50–59; 33% for 60–69; 37% for 70–79
						Japan By age: 22% for 40–49; 25% for 50–59; 36% for 60–69; 49% for 70–79
						China By age: 19% for 40–49; 24% for 50–59; 33% for 60–69; 49% for 70–79
						Taiwan By age: 11% for 40–49; 26% for 50–59; 37% for 60–69; 49% for 70–79
						Korea By age: 12% for 40–49; 36% for 50–59; 52% for 60–69; 90% for 70–79
						Philippines By age: 44% for 40–49; 24% for 50–59; 52% for 60–69; 64% for 70–79
						Thailand By age: 20% for 40–49; 31% for 50–59; 50% for 60–69; 53% for 70–79
						Singapore By age: 14% for 40–49; 18% for 50–59; 44% for 60–69; 53% for 70–79
						Pakistan By age: 14% for 40–49; 33% for 50–59; 40% for 60–69; 51% for 70–79
						India By age: 25% for 40–49; 37% for 50–59; 37% for 60–69; 50% for 70–79
Chicharro-Molero *et al*.	Spain	1998	1173 men aged >40 yrs who lived in Andalusia	Population based, cross sectional, self-administered survey with uroflowmetry	Moderate or severe symptoms (total score >7) on the IPSS, maximum flow rate <15 ml/s and prostate size >30 gm	Overall: 24.9% By age: 10.6% for 40–49; 21.0% for 50–59; 28.5% for 60–69; 45.0% for >70
Trueman *et al*.	England, Scotland, Wales	1999	1500 men aged >50 yrs stratified by age (74%)	Population based, cross sectional, postal questionnaire	Moderate or severe symptoms (total score >7) on the IPSS	Overall: 41% By age: 29% for 50–59; 38% for 60–69; 41% for 71–80; 51% for >80
Blanker *et al*.	Netherlands	2000	3924 men aged 50–75 yrs from Krimpen (50%)	Clinic based, cross sectional, self-administered survey with physical examination	Variable depending on definition (1) IPSS >7; (2) IPSS >7 & prostate volume >30 mL; (3) IPSS >7, prostate volume >30 mL & Q_max_ < 15 mL/sec; (4) IPSS >7, prostate volume >30 mL & Q_max_ < 10 mL/sec; or (5) IPSS >7, prostate volume >20 mL & Q_max_ < 15 mL/sec	(1) Overall: 25% By age: 21% for 50–54; 19% for 55–59; 24% for 60–64; 31% for 65–69; 37% for 70–78
						(2) Overall: 14% By age: 7% for 50–54; 7% for 55–59; 15% for 60–64; 22% for 65–69; 28% for 70–78
						(3) Overall: 12% By age: 6% for 50–54; 6% for 55–59; 13% for 60–64; 20% for 65–69; 27% for 70–78
						(4) Overall: 9% By age: 4% for 50–54; 4% for 55–59; 9% for 60–64; 14% for 65–69; 23% for 70–78
						(5) Overall: 20% 8 By age: 14% for 50–54; 14% for 55–59; 20% for 60–64; 26% for 65–69; 32% for 70–7
Teh *et al*.	Malaysia	2001	578 men aged >50 yrs in Kuala Lumpur	Community based, cross sectional, survey	Prostate volume >20cc on transrectal ultrasonography with IPSS total score >7	Overall: 39.3% By age: 35.0% for 50–59; 43.0% for 60–69; 52.6% for >70
Berges *et al*.	Germany	2001	8973 men aged 50–80 from Herne (60.2%)	Community based, cross sectional, survey	IPSS total score >7	Overall: 29.3% By age: 21.5% for 50–59; 27.1% for 60–69; 38.2% for >70
Lee *et al*.	Korea	2005	1298 men aged >65 yrs from Anyang	Population based, cross sectional, interview	IPSS total score >7	Overall: 19.7% By age: 17.6% for 65–69; 22.2% for 70–74; 21.0% for 75–79; 20.0% for 80–84
Roehrborn *et al*.	USA	2006	>2000 men aged 50–79 yrs from national sample	Population based, cross sectional, telephone interview	Moderate or severe symptoms (total score >7) on the AUA-SI	Overall: 25% By age: 14% for 50–59; 30% for 60–69; 40% for 70–79
Naslund *et al*.	USA	2007	All male >50 yrs from six regions (California, Georgia, Maryland, Ohio, Texas and Wyoming)	Physician clinic based, cross sectional, self-administered questionnaire and physical examination	IPSS total score >7	Overall: 42% By age: 33% for 50–59; 50% for 60–69; 46% for ≥70
Kristal *et al*.	USA	2007	5,667 men aged >50 yrs from the Prostate Cancer Prevention Trial	Prospective, cohort study, self-administered questionnaire and physician clinic visit	Receipt of treatment or report of 2 IPSS total score >14. Severe BPH is defined as treatment or 2 IPSS >20	Overall: 18.4% By age: 14.2% for 50–59; 17.6% for 60–64; 23.3% for ≥65
Safarinejad *et al*.	Iran	2008	8,466 men aged >40 yrs from 30 counties in Iran	Community based, cross sectional, 2 stage sampling, interview and physical examination	Persian translated of IPSS total score >7, Qmax <15 ml/s and prostate size >30 g	Overall: 23.8% By age: 1.2% for 40–49; 18.4% for 50–59; 26.8% for 60–69; 36.0% for ≥70
Huh *et al*.	Korea	2012	553 men aged >50 yrs from Jeju Island	Community based, cross sectional, survey with physical examination	Korean translation of IPSS total score >7 with prostate volume >30 g estimated from transrectal ultrasound	Overall: 21.0% By age: 11.6% for 50–59; 18.1% for 60–69; 30.8% for 70–79; 50.8% for ≥80
						Age adjusted: 16.8% for 50–59; 21.7% for 60–69; 24.4% for 70–79; 28.1 for ≥80
Chokkalingam *et al*.	Ghana	2012	1049 men aged 50–74 yr from Accra (93.4%)	Community based, cross sectional, interview and physical examination	Prostate symmetrically enlarged (estimated 30 cm^3^ or larger) with total IPSS score >7	Overall: 13.3% By age: 8.9% for 50–59; 17.1% for 60–69; 21.8% for 70–74
Goh *et al*.	Korea	2015	779 men aged >40 yrs from Yangpyeong Country	Community based, cross sectional, survey with physical examination	IPSS total score >7 and prostate volume >25 mL on transrectal ultrasound	Overall: 20.0% By age: 4.4% for 40–49; 10.9% for 50–59; 22.2% for 60–69; 26.6% for >70
Arafa *et al*.	Saudi Arabia	2015	1,851 men aged >40 yrs from Riyadh	Hospital outpatient, cross-sectional, interview and physical examination	Arabic version of IPSS with total score >7, digital rectal examination and ultrasound	Overall: 31.7% By age: 36.6% for <50; 26.4% for 50–60; 34.5% for 61–70; 46.4% for >70
Egen *et al*.	USA	2015	Data on men aged >40 years from the 2001–2008 National Health and Nutrition Examination Survey	Population based, cross sectional, survey	Self-reported of physician diagnosed enlarged prostate and/or BPH medication. Unrecognised BPH was defined as urinary symptoms such as incomplete urination and/or difficulty in urination	Overall recognised: 16.5% Overall unrecognised: 9.6%
						By age: 19.9% for 40–59; 37.0% for 60–69; 50.5% for 70–79; 58.2% for ≥80
Da *et al*.	China	2015	Male residents >50 years old in 5 communities in Shanghai	Community based, cross sectional, interview with physical examination	Physician diagnosed based upon patient history of LUTS, urinalysis, DRE, ultrasound and uroflowmetry.	Overall: 12.0% By age: 5.2% for 50–59; 14.0.% for 60–69; 22.9% for 70–79; 25.0% for ≥80

AUA– American Urological Association.

BPH- benign prostatic hyperplasia.

DRE – Digital rectal examination.

IPSS – International prostate symptom score.

LUTS – Lower urinary tract symptom.

Most of the included studies in the current review were cross sectional which had gathered data prospectively from surveys or interviews. These surveys can cover the whole country, as in the case of study with Egan and colleagues^38^, or a specific geographical area within the country, such as Shanghai in China^39^. The case definition of LUTS/BPH varied substantially across studies, depending on the criteria used. Ten studies used both objective as well as subjective parameters including measurement of prostate size as well as uroflowmetry while seventeen studies relied solely upon the presence of moderate to severe LUTS. Four studies only used objective measurements as the case definition for LUTS/BPH. The most common tool used for measuring severity of LUTS was the AUA -SI or IPSS, which was used in 24 studies, while 2 studies used a urinary dysfunction questionnaire and 1 used the Madsen questionnaire. When using the modified Newcastle-Ottawa quality assessment criteria, 2 studies received the maximum 5 points, 11 received 4 points, 7 received 3 points, 6 received 2 points and 5 received 1 point (Supplementary Table S1).

### Epidemiology

In general, the prevalence of BPH increases with increasing age, with the highest prevalence in participants aged 70 and above. The median point prevalence was 25.2% and the 10% and 90% quartiles ranged from 19.0% to 37.9%. The highest prevalence of BPH was reported by Naslund and colleagues^31^ who surveyed patients from their clinic from 6 US states in 2007 while the lowest prevalence was found in Da and colleagues in Shanghai, China^39^. Meta-analysis of 30 studies using a random effects model yielded a summary prevalence of 26.2% (16,437/76,246 individuals; 95% CI: 22.8–29.8%). However, a high level of heterogeneity was observed (*I* ^2^ = 99.2%, Q-value = 3493.89, τ = 0.01, p < 0.01). Serial exclusion of each study in the sensitivity analysis demonstrated that no individual study influenced the overall prevalence by more than 1% (Supplementary Table S2).

To provide a range of BPH prevalence estimates due to the methodologically diverse studies, estimates were stratified according to diagnostic criteria and study level characteristics. When evaluated by BPH diagnostic criteria, summary prevalence estimates ranged from 26.2% for studies that used only objective measurements (798/2,837 individuals, 95% CI, 22.8–29.6%, *Q* = 3.60, τ^2^ = 0.001, *I* ^*2*^ = 44.5%) to 28.8% when using only subjective questionnaires such as the AUA-SI or the IPSS (8,417/28,421 individuals, 95% CI, 25.2–32.3%, *Q* = 654.93, τ^2^ = 0.006, *I* ^*2*^ = 97.7%). In the 11 studies that used both objective and subjective questionnaires, it yielded a lower prevalence estimates of 22.6% (7,221/44,723 individuals, 95% CI: 18.4–26.9%, Q = 877.60, τ^2^ = 0.005, *I* ^2^ = 98.9%).

Higher prevalence estimates were found among studies conducted in the United States versus elsewhere (3,765/14,284, 29.2% [95% CI, 22.3% to 36.1%] vs 12,672/61,962, 25.5% [95% CI, 21.5% to 29.4%]; *Q* = 0.85, *P* = 0.36), but this was not statistically significant. Similarly, no statistically significant difference in prevalence estimates were noted when stratified between rural, urban or mixed populations (Q = 0.58, p = 0.90), comparing respondent representativeness (Q = 0.04, p = 0.85), cohort sample size (Q = 0.01, p = 0.99) or study quality (Q = 0.22, p = 0.64). However, the prevalence rate was much lower in the study conducted among Africans compared to those conducted among Asian or Caucasians (Q = 101.34, p < 0.01). In the meta-regression analysis, none of the covariates analysed were significantly associated were associated with heterogeneity of prevalence estimates (Table 2).

**Table 2:** Association between study variables and BPH prevalence estimates.

Study characteristics		Univariate regression-model			
		Estimated prevalence difference	Standard error	95% CI	p-value
Sample size					
<1000	Reference				
≥1000	−0.08%	2.84	−5.91	5.76	0.98
Study location					
Urban	Reference				
Rural	−1.46%	4.79	−11.42	8.50	0.76
Mixed	0.05%	4.13	−8.06	9.12	0.90
Origin of sample population					
Community	Reference				
Population	1.91%	3.09	−4.43	8.24	0.54
Clinic	4.63%	4.53	−4.94	13.66	0.35
BPH definition criteria					
Laboratory/Physical examination	Reference				
Symptom only	2.30%	4.58	−7.11	12.70	0.62
Symptom with physical examination	−3.80%	4.82	−13.68	6.09	0.44
Study continent					
North America	Reference				
Europe	−3.60%	4.01	−11.86	4.66	0.36
Asia	−2.99%	4.02	−11.26	5.29	0.46
Australia/New Zealand	−8.29%	8.65	−26.11	9.52	0.35
Africa	−8.52%	8.52	−26.50	8.59	0.30
Data collection method					
Survey	Reference				
Interview	−1.67%	3.54	−8.96	5.62	0.64
Postal	4.61%	5.93	−7.59	16.82	0.44
Database review	5.51%	8.09	−11.16	22.17	0.50
Telephone interview	1.09%	5.97	−13.38	11.21	0.86
Race/Ethnicity					
Caucasian	Reference				
Asian	−1.21%	3.33	−8.06	5.64	0.72
Mixed	1.32%	3.89	−6.68	9.32	0.74
African	−6.32%	8.17	−23.12	10.48	0.45

### Age specific prevalence

**Figure 2:**
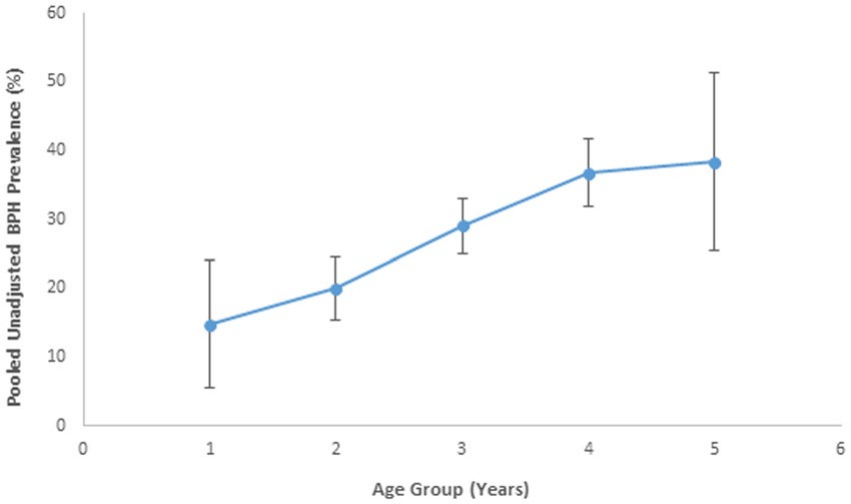
Unadjusted benign prostatic hyperplasia prevalence based across different age groups.

Of the total 31 studies, only 25 studies reported age-specific stratified data for analysis. Grouped summary data showed that there was an increasing prevalence of LUTS/BPH with age, with a pooled prevalence of 14.8%, 20.0%, 29.1%, 36.8% and 38.4% for age groups of 40–49 years, 50–59 years, 60–69 years, 70–79 years and 80 years and above respectively (Fig. 2), but there was a high level of heterogeneity.

### Prevalence rates across the years

**Figure 3:**
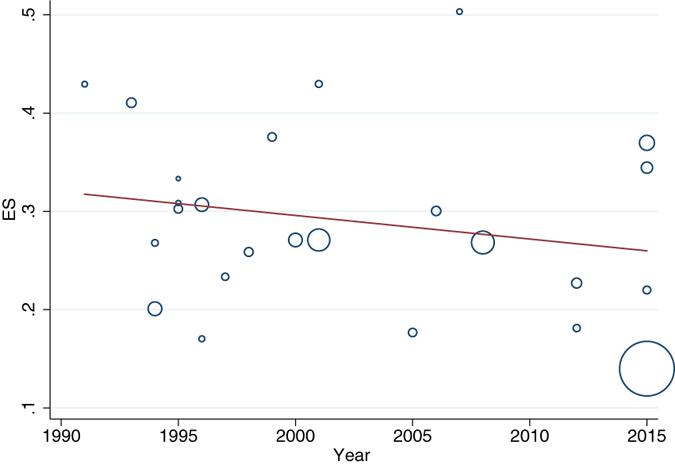
Benign prostatic hyperplasia prevalence by year of data collection.

The prevalence rates of BPH for the survey years of 1990–1999, 2000–2009, and 2010 till present were 26.6%, 27.8% and 22.8% respectively. The prevalence rates were not significantly change with baseline survey year (slope = −0.24% per calendar year increase; 95% CI: −0.71 to 0.23; p = 0.30; Fig. 3). No significant interaction was detected when we tested the interactive effects with different study characteristics, suggesting that prevalence estimates were not affected by time in geographical regions or study methods.

### Sensitivity analyses

Sensitivity analyses showed that there was very little difference in prevalence estimates when studies were excluded sequentially, or stratified by sample size and study quality. However, visual inspection of funnel plot was asymmetrical, suggesting that there was some evidence for bias due to small-study effects (Supplementary Fig. S1).

## Discussion

BPH is a common condition that affects millions of men worldwide. In this study, we systematically reviewed studies to estimate the prevalence of BPH. We found that the pooled prevalence was 26.2% (95% CI: 22.8–29.6%), with estimates differing across studies, because of different BPH definitions, survey methods, response options, geographical locations and sample populations. Despite this, we found that the prevalence of BPH increases as patient age increase, from 14.8% in younger males aged 40 to 36.8% in males aged 80 and above. In most traditional prevalence studies, estimates are usually obtained based upon the population living in a specified area. However, this introduces a selection bias to the study as these boundaries (e.g., cities, county or even nations) may be suboptimal for the detection of variation of disorder between or even within a specific population. Similarly, factors such as age or even migration patterns can influence these estimate. The prevalence of BPH is also thought to vary across difference ethnicities, urban/rural settings and countries. Some of these differences may be attributable to the methodological differences but these variation does exist even in a multinational study which had identical BPH definitions, survey and response format. We also found that there was considerable variability even within studies in the same country. These differences may result from many different sociocultural and environmental factors that can affect prostate health, in addition to genetic factors.

Some potential reasons for the marked difference in estimated prevalence could be due to the methodological differences used by different studies. For example, in the study by Trueman and colleagues^25^, the presence of BPH was assessed using a self-administered questionnaire. In contrast, a study by Goh and associates^40^ in Korea reported a prevalence rate of 20%. Participants were surveyed by trained investigators and prostate disease was assessed by a physical examination and measurement of prostate volume and serum prostate specific antigen. The current AUA guideline defines LUTS/BPH as the presence of voiding and/or storage symptoms^41^. The absence of a specific, universally accepted operational criterion has led to a diversity of definition and variance in incidence estimates. These methodological differences are in part, likely to account for some of the differences in findings between studies.

The definition of BPH has been problematic due to the variation in case definition used by different studies. In many older studies identified, BPH is commonly described as a chronic urinary symptom experienced by elderly men. However, some studies had defined BPH using radiographically determined prostate enlargement, while others had used the definition of decreased in urinary flow rates, urinary symptoms and in some cases physician-diagnosed BPH. However, the most commonly used measures by which BPH is diagnosed are the AUA-SI and its internationally validated counterpart, the IPSS. This heterogeneity in BPH definition is thought to account for some differences in BPH prevalence rates.

The significant heterogeneity observed in this study led to a subgroup analysis as well as a meta-regression analyses to determine the potential sources. Effect estimates were similar when studies were grouped according to patient characteristics, suggesting that much of the heterogeneity remains unexplained. As with most meta-analysis of summary data, this study failed to identify the main source of heterogeneity. However, the meta-regression approach has several drawbacks. Firstly, it uses only the average value of a particular characteristic rather than individual values, thus decreasing the power to detect associations. Secondly, the meta-analysis also depended on the availability of published data, and more often than not, most of the methodology is incomplete.

This study has several strengths. To our knowledge, this is the first meta-analysis that attempts to examine and summarise the global prevalence of BPH. This systematic review conforms to the guidelines of Meta-Analysis of Observational Studies in Epidemiology (MOOSE)^42^ recommendations. Another strength of this study include a comprehensive and broad search strategy, as well as relevance of finding to clinical practice and research.

This study has some limitations. Firstly, as mentioned, the differences in study methodology and population may have considerable effects on the results. These effect contribute to the substantial variability in reported BPH rates, and it is difficult, if not impossible to separate these effects from the true geographical, cultural, economic and psychosocial differences. Secondly, we also restricted our search to only articles published in English, and thus we may have missed some important data. We did not search “grey literature”, as we felt that most of these data would not be sufficiently informative^43^. The current study could not take in consideration other risk factors associated with LUTS/BPH including diet, diabetes, or even body mass index which has substantially changed over the past 3 decades. Similarly, this study could not account for the variation in criteria of LUTS/BPH that has been revised. As such, inclusion of older studies may have led to an underestimation of the prevalence rates.

In summary, the current review provides a benchmark on the prevalence estimates for BPH. However, the wide range of prevalence estimates and case definition suggest that a standard criteria needs to be applied given the importance of understanding the prevalence of BPH and its implication on public health given the increasingly rapid growth of elderly worldwide. Additional research is needed in various areas especially on economic parameters.

## Methods

### Search strategy and selection criteria

We performed a literature search up until 31 July 2016 using a combination of search terms (Appendix 1) on the following database: Pubmed, EMBASE, Cinahl plus, AMED, CENTRAL and Web of Science for articles describing the prevalence of BPH among males. Keywords used include: prevalence, incidence, prostate enlargement, benign prostate hyperplasia, benign prostatic hypertrophy, bladder outlet obstruction and lower urinary tract symptoms. Two authors independently (SWHL & EMCC) reviewed the records to identify for potentially studies and full text of studies were retrieved if necessary. We also manually search bibliographies of included studies and any relevant review articles for additional references. In the event of multiple publications of identical data, the most informative version of the study was used. Any discrepancies were resolved by open discussion.

### Definition

While the term BPH is correctly defined as histopathological hyperplastic changes in the prostate^41^, most studies and clinicians commonly use the term to describe a clinical syndrome that comprised of LUTS, prostatic enlargement and bladder outlet obstructions. In this study, we used the case definition for BPH as stated in the study. In the event that this was not stated, BPH was defined as the presence of moderate to severe LUTS, and used a cut off score of >7 for the American Urological Association Symptom Index (AUA-SI)^44^ or the shorter version International Prostate Symptom Score (IPSS). Countries were regarded as industrialised if they fell within the high income definition as defined by the World Health Organisation.

### Data extraction and assessment

Two authors separately extracted the studies using a standardised extraction template, including study level characteristics (e.g., urban/rural, study design, year study was conducted, definition of BPH and data collection methods) as well population characteristics (e.g., age-specific rates and ethnicity/race). Study authors were contacted for data clarification if necessary. The methodological quality of each study was judged using a modified version of the Newcastle-Ottawa Scale^45^, which includes 4 criteria namely, sample representativeness and size, comparability between respondents and non-respondents, ascertainment of BPH symptoms and statistical quality. Studies were judged to be low risk of bias if they had a minimum score of 3 points of the maximum 5 points.

### Statistical analysis

We conducted a meta-analysis of incidence data and pooled the estimates and 95% confidence intervals (CI) using the metaprop command developed by the Unit Cancer Epidemiology in Brussel^46^, and used the random-effects model since we expect the presence of heterogeneity. We subsequently conducted a subgroup analysis, and stratified the studies according to study geographic regions, number of cases of BPH, tools used to detect BPH as well as age groups as reported by the study. Potential small study publication bias was assessed using the Begg & Eggers test, as well as visual inspection of the funnel plot. Between studies heterogeneity was assessed using I^2^ and Cochran’s Q method. In the event of substantial heterogeneity, a random-effects meta-regression analysis was conducted to determine the effects of variables such as population demographics, study characteristics and indicators of error or bias on prevalence estimates. Any factor(s) which was significant in the univariate analysis were included into a multiple regression model. We also performed several sensitivity analyses to assess how our primary estimates differed when we excluded studies sequentially as well as studies with lower methodological quality such as those with poor sampling methods or sample sizes less than 1000 participants. We also assessed the possibility of change in prevalence patterns over time by examining prevalence rates across different study years. All analyses was conducted using Stata version 13.0 (StataCorp, College TX).

## Electronic supplementary material

Supplementary Information
