# Supplementary material for: The global burden of lower urinary tract symptoms suggestive of benign prostatic hyperplasia: A systematic review and meta-analysis
Source: Sci Rep. 2017 Aug 11;7:7984. doi: 10.1038/s41598-017-06628-8 (PMC5554261; doi:10.1038/s41598-017-06628-8)
Supplement: Supplementary file 1 — Supplementary Information [file 41598_2017_6628_MOESM1_ESM.docx]

# The global burden of lower urinary tract symptoms suggestive of benign prostatic hyperplasia: A systematic review and meta-analysis

Shaun Wen Huey Lee* , Esther Mei Ching Chan, Yin Key Lai

# Keywords

1. Benign prostate hyperplasia
2. Urinary retention
3. Lower urinary tract symptoms
4. Prostate enlargement
5. OR/1-4
6. Epidemiology
7. Prevalence
8. Incidence
9. Lifetime prevalence
10. OR/6-9
11. #5 AND #10

**Figure S1: Publication bias**

Begg’s Test p-value = 0.32

# Modified Newcastle-Ottawa scoring guide.

(**1) Representativeness of the sample:**

1 point: Population surveyed from multiple sites.

0 points: Population contained a single site, clinic patients.

**(2) Sample size:**

1 point: Sample size was greater than 1000 participants.

0 points: Sample size was less than 1000 participants or a convenience sample.

**(3) Non-respondents:**

1 point: Comparability between respondent and non-respondent characteristics was

established, and the response rate was satisfactory.

0 points: The response rate was unsatisfactory, the comparability between respondents

and non-respondents was unsatisfactory, or there was no description of the response rate

or the characteristics of the responders and the non-responders.

**(4) Ascertainment of benign prostatic hyperplasia:**

1 point: Validated measurement tool using a validated cutoff score.

0 points: Non-validated measurement tool, or validated measurement tool with non-valid

cutoff score, or self-reported prevalence (scored as such due to its low specificity).

**(5) Quality of descriptive statistics reporting:**

1 point: Reported descriptive statistics to describe the population (*e.g.*, age, sex) with

proper measures of dispersion (*e.g.*, standard deviation, standard error, range).

0 points: Descriptive statistics were not reported, were incomplete, or did not include

proper measures of dispersion.

**Legend:** This scale, the scoring of which ranges from 0 to 5, assesses quality in several domains:

sample representativeness and size, comparability between respondents and non-respondents,

ascertainment of depressive symptoms, and statistical quality. Studies were judged to be of low

risk of bias (≥3 points) or high risk of bias (<3 points).

**Table S1: Modified Newcastle Ottawa Scale**

| Study | Year | Representativeness of exposed cohort | Sample size | Non-respondent | Ascertainment of BPH | Descriptive stats reporting | Score |
| --- | --- | --- | --- | --- | --- | --- | --- |
| Sommer | 1990 | 1 | 0 | 0 | 0 | 0 | **1** |
| Garraway | 1991 | 0 | 0 | 0 | 1 | 0 | **1** |
| McKelvie | 1993 | 0 | 1 | 0 | 1 | 0 | **2** |
| Chute | 1993 | 1 | 1 | 1 | 0 | 1 | **4** |
| Sagnier | 1994 | 1 | 1 | 0 | 1 | 1 | **4** |
| Norman | 1994 | 1 | 0 | 1 | 1 | 1 | **4** |
| Hunter | 1994 | 1 | 1 | 1 | 0 | 1 | **4** |
| Bosch | 1995 | 0 | 0 | 0 | 0 | 1 | **1** |
| Tsukamoto | 1995 | 0 | 0 | 1 | 1 | 0 | **2** |
| Nacey | 1995 | 1 | 0 | 0 | 1 | 1 | **3** |
| Ukimura | 1996 | 1 | 0 | 0 | 1 | 0 | **2** |
| Hunter | 1996 | 1 | 1 | 1 | 1 | 1 | **5** |
| Simpson | 1996 | 1 | 0 | 1 | 1 | 1 | **4** |
| Homma | 1997 | 1 | 1 | 0 | 1 | 1 | **4** |
| Lee | 1997 | 1 | 0 | 1 | 1 | 0 | **3** |
| Chicharro-Molrto | 1998 | 1 | 1 | 0 | 1 | 1 | **4** |
| Trueman | 1999 | 1 | 1 | 0 | 0 | 1 | **3** |
| Blanker | 2000 | 1 | 1 | 1 | 1 | 1 | **5** |
| Teh | 2001 | 0 | 1 | 0 | 1 | 1 | **3** |
| Berges | 2001 | 1 | 1 | 0 | 1 | 1 | **4** |
| Lee | 2005 | 1 | 1 | 0 | 1 | 1 | **4** |
| Roehrborn | 2006 | 1 | 1 | 0 | 0 | 1 | **3** |
| Naslund | 2007 | 1 | 0 | 0 | 1 | 0 | **2** |
| Kristal | 2007 | 0 | 1 | 0 | 0 | 1 | **2** |
| Safarinejad | 2008 | 1 | 1 | 0 | 1 | 1 | **4** |
| Huh | 2012 | 0 | 0 | 0 | 1 | 1 | **2** |
| Chokkalingam | 2012 | 1 | 0 | 0 | 1 | 1 | **3** |
| Egan | 2015 | 1 | 1 | 0 | 0 | 1 | **3** |
| Da | 2015 | 0 | 1 | 0 | 0 | 0 | **1** |
| Goh | 2015 | 0 | 0 | 1 | 1 | 1 | **3** |
| Arafa | 2015 | 0 | 1 | 1 | 1 | 1 | **4** |

**Table S2: Study results after serial exclusion of studies**

| **First author study omitted** | **Prevalence (%)** | **Lower 95% CI** | **Upper 95% CI** |
| --- | --- | --- | --- |
| Garraway, 1991 | 26.2 | 22.7 | 29.7 |
| Chute, 1993 | 25.9 | 22.5 | 29.4 |
| McKelvie, 1993 | 26.2 | 22.7 | 29.7 |
| Norman, 1994 | 26.3 | 22.8 | 29.8 |
| Hunter, 1994 | 26.4 | 22.9 | 29.9 |
| Sagnier, 1994 | 26.6 | 23.1 | 30.2 |
| Bosch, 1995 | 26.1 | 22.6 | 29.6 |
| Tsukamoto, 1995 | 25.9 | 22.4 | 29.3 |
| Nacey, 1995 | 26.4 | 22.9 | 29.9 |
| Ukimura, 1996 | 26.2 | 22.7 | 29.7 |
| Hunter, 1996 | 26.1 | 22.6 | 29.5 |
| Simpson, 1996 | 26.4 | 22.9 | 29.9 |
| Lee, 1997 | 26.3 | 22.8 | 29.9 |
| Homma, 1997 | 25.9 | 22.3 | 29.1 |
| Chicharro-Molero, 1998 | 26.4 | 22.9 | 29.9 |
| Trueman, 1999 | 25.7 | 22.3 | 29.1 |
| Blanker, 2000 | 26.4 | 22.9 | 29.8 |
| Teh, 2001 | 25.8 | 22.3 | 29.2 |
| Berges, 2001 | 26.1 | 22.7 | 29.9 |
| Lee, 2005 | 26.4 | 22.9 | 29.9 |
| Roehrborn, 2006 | 26.2 | 22.7 | 29.7 |
| Naslund, 2007 | 25.7 | 22.2 | 29.1 |
| Kristal, 2007 | 26.5 | 22.8 | 30.2 |
| Safarinejad, 2008 | 26.3 | 22.6 | 30.0 |
| Huh, 2012 | 26.4 | 22.9 | 29.9 |
| Chokkalingam, 2012 | 26.4 | 22.9 | 29.9 |
| Goh, 2015 | 26.2 | 22.5 | 29.9 |
| Egan, 2015 | 26.0 | 22.6 | 29.4 |
| Da, 2015 | 26.7 | 24.2 | 29.1 |
| Arafa, 2015 | 26.0 | 22.5 | 29.5 |
|  |  |  |  |
| **Pooled estimate** | **26.2** | **22.8** | **29.6** |
